# The Thyroid Hormone Receptors Modulate the Skin Response to Retinoids

**DOI:** 10.1371/journal.pone.0023825

**Published:** 2011-08-17

**Authors:** Laura García-Serrano, María Ana Gomez-Ferrería, Constanza Contreras-Jurado, Carmen Segrelles, Jesus M. Paramio, Ana Aranda

**Affiliations:** 1 Instituto de Investigaciones Biomédicas, Consejo Superior de Investigaciones Científicas and Universidad Autónoma de Madrid, Madrid, Spain; 2 Centro de Investigaciones Energéticas, Medioambientales y Tecnológicas, Madrid, Spain; Laboratoire Arago, France

## Abstract

**Background:**

Retinoids play an important role in skin homeostasis and when administered topically cause skin hyperplasia, abnormal epidermal differentiation and inflammation. Thyroidal status in humans also influences skin morphology and function and we have recently shown that the thyroid hormone receptors (TRs) are required for a normal proliferative response to 12-*O*-tetradecanolyphorbol-13-acetate (TPA) in mice.

**Methodology/Principal findings:**

We have compared the epidermal response of mice lacking the thyroid hormone receptor binding isoforms TRα1 and TRβ to retinoids and TPA. Reduced hyperplasia and a decreased number of proliferating cells in the basal layer in response to 9-cis-RA and TPA were found in the epidermis of TR-deficient mice. Nuclear levels of proteins important for cell proliferation were altered, and expression of keratins 5 and 6 was also reduced, concomitantly with the decreased number of epidermal cell layers. In control mice the retinoid (but not TPA) induced parakeratosis and diminished expression of keratin 10 and loricrin, markers of early and terminal epidermal differentiation, respectively. This reduction was more accentuated in the TR deficient animals, whereas they did not present parakeratosis. Therefore, TRs modulate both the proliferative response to retinoids and their inhibitory effects on skin differentiation. Reduced proliferation, which was reversed upon thyroxine treatment, was also found in hypothyroid mice, demonstrating that thyroid hormone binding to TRs is required for the normal response to retinoids. In addition, the mRNA levels of the pro-inflammatory cytokines TNFα and IL-6 and the chemotactic proteins S1008A and S1008B were significantly elevated in the skin of TR knock-out mice after TPA or 9-cis-RA treatment and immune cell infiltration was also enhanced.

**Conclusions/significance:**

Since retinoids are commonly used for the treatment of skin disorders, these results demonstrating that TRs regulate skin proliferation, differentiation and inflammation in response to these compounds could have not only physiological but also therapeutic implications.

## Introduction

The skin, which protects organisms from the external environment, is comprised of a stratified epithelium, the epidermis, separated by a basement membrane from the underlying connective tissue, the dermis. Skin homeostasis depends upon a strict control of epidermal proliferation and differentiation. Keratinocytes in the basal layer of the epidermis are able to proliferate, differentiating as they move towards the skin surface progressing through three layers, stratum spinosum, stratum granulosum, and stratum corneum in which eventually cells slough off the skin. Skin layers correspond to different differentiation stages and they express specific markers: basal-layer keratinocytes specifically express keratin (K) 5 and K14; spinous cells express K1 and K10; and filaggrin, loricrin and transglutaminase 3 are produced in the granular layer [1].

Retinoids, the biologically active derivatives of vitamin A, regulate important biological processes, such as development, control and maintenance of homeostasis, cell growth, differentiation and death [2]. Retinoids play an important role in skin homeostasis and are widely used in cosmetics and in the treatment of skin disorders [3]. In the skin, topical application of retinoic acid (RA) generates epidermal hyperplasia that results from hyperproliferation of basal keratinocytes leading, upon their vectorial migration towards the skin surface, to thickening of the differentiated suprabasal layers [3]. RA treatment also alters epidermal cell differentiation causing parakeratosis [4] and reducing expression of granular markers such as loricrin [4], [5], [6], [7], [8], [9], and causes skin irritation with infiltration of immune cells [10], [11]. Paradoxically, RA induces opposite responses in keratinocytes depending on whether they are studied *in vivo* or cultured *in vitro*, since RA treatment induces keratinocyte growth-arrest *in vitro* [12], while stimulating keratinocyte proliferation *in vivo*.

Retinoids exert their action by binding to retinoic acid receptors (RARα, -β, and –γ), whose inactivation has a profound effect in skin structure and function [13], [14]. RARs regulate gene expression by binding as heterodimers with other members of the nuclear receptor superfamily, the retinoid-X-receptors (RXRα, -β, and –γ), to response elements located in the regulatory regions of their target genes. However, retinoid receptors can modulate gene expression through other mechanisms, including competition of RARs with RXRs for binding to other heterodimerization partners and transrepression of the activity of other transcription factors [2], [15], [16]. RARs can be activated by all-trans retinoic acid (all-trans-RA) and its stereoisomer, 9-cis retinoic acid (9-cis-RA), whereas RXRs are only activated by 9-cis-RA. However, the presence of 9-cis-RA as a true endogenous ligand, in the absence of excessive retinoid intake, is questionable and some as of yet unidentified ligand for RXR could be involved in its activity in that tissue [17].

Similar to RA, the thyroid hormones exert their effects through binding to nuclear receptors (TRα and TRβ), which are known to be expressed in the skin [18], [19], [20], [21]. TRs interact with thyroid hormone responsive elements (TREs), as heterodimers with RXRs [22]. In hypothyroid humans the skin is cool and dry with a pasty appearance, the epidermis is thin and hyperkeratotic, alopecia may develop, and there is diffuse myxedema [23], [24], [25], [26], showing that thyroidal status influence skin morphology and function. In addition, topical application of the thyroid hormone triiodothyronine (T3) stimulates epidermal proliferation and dermal thickening, in both mice and rats [27], [28]. A TRα mutant mice has been found to display retarded hair growth [29], and using genetically modified mice we have recently observed that effects of thyroid hormone on cell proliferation are mediated through interactions with both TRα and TRβ [30]. We found reduced keratinocyte proliferation and decreased hyperplasia in response to topical application of 12-*O*-tetradecanolyphorbal-13-acetate (TPA), a well-known model for induction of skin hyperproliferation and inflammation, in the epidermis of knock-out mice lacking the thyroid hormone binding isoforms TRα1 and TRβ. Both receptor isoforms appear to play overlapping functional roles, since mice lacking individually TRα1 or TRβ also present a proliferative defect but not as marked as that found in double KO mice. Similar results were obtained in hypothyroid animals, showing the important role of the liganded TRs on epidermal proliferation. In addition, KO animals display augmented expression of chemokines and proinflammatory cytokines, demonstrating that TRs can also act as endogenous inhibitors of skin inflammation[30].

In this work we have compared the epidermal response to retinoids and the tumor promoter in hypothyroid and TR KO mice. We found that liganded TRs are also required for a normal response to retinoids. Skin hyperplasia, proliferation of keratinocytes and expression of keratins 5 and 6 are reduced in TR-deficient mice treated with 9-cis-RA or TPA. As expected, the retinoid caused parakeratosis and a decreased expression of differentiation markers such as keratin 10 or loricrin, and these responses were altered in the TR KO mice in which parakeratosis was inhibited and expression of these markers was further reduced, indicating that these receptors can play a role in both early and terminal keratinocyte differentiation. Finally, increased levels of transcripts coding for inflammatory cytokines and chemotactic proteins were obtained in TR-deficient mice treated with either TPA or 9-cis-RA, suggesting an increased inflammatory response. This was further demonstrated by the finding that infiltration of immune cells was increased in treated skins of TR KO mice.

## Results

### Reduced proliferative response to retinoids in mice lacking thyroid hormone receptors

**Figure 1:**
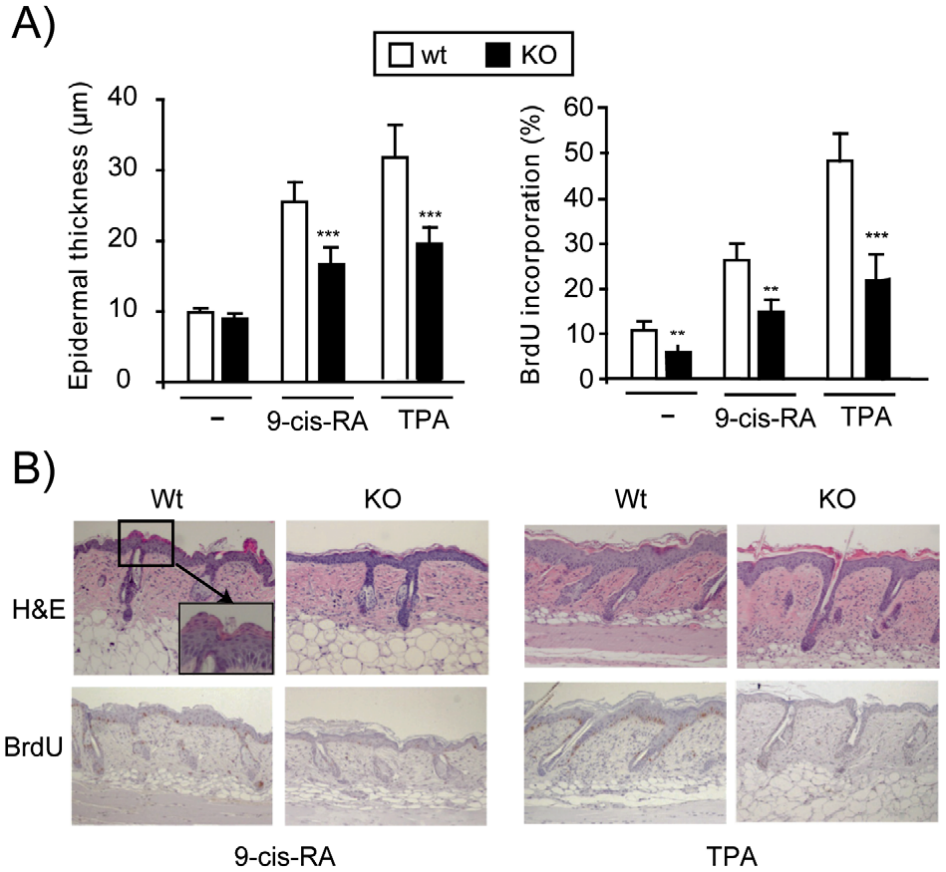
Reduced skin proliferation after 9-cis-RA and TPA treatment in TR-deficient mice. A) Dorsal skins of wild-type mice (Wt) and knock-out mice lacking TRα1 and TRβ (KO), were treated topically with vehicle (acetone), 9-cis-RA or TPA. Morphometric analysis were performed to determine epidermal thickness (left panel) and proliferation rate in the basal layer was quantitated and is represented as the percentage of positive BrdU cells *vs.* total cells (right panel). Data are shown as mean values ± SE, and asterisks denote statistically significant differences relative to Wt mice (*, *P*<0.05; ***, *P*<0.001). B) The upper panels show representative H&E staining of 9-cis-RA and TPA treated mice. The magnification in wild-type mice shows parakeratosis induced by 9-cis-RA, which was absent in KO mice. The lower panels show representative BrdU immunostaining in the same groups.

**Figure 2:**
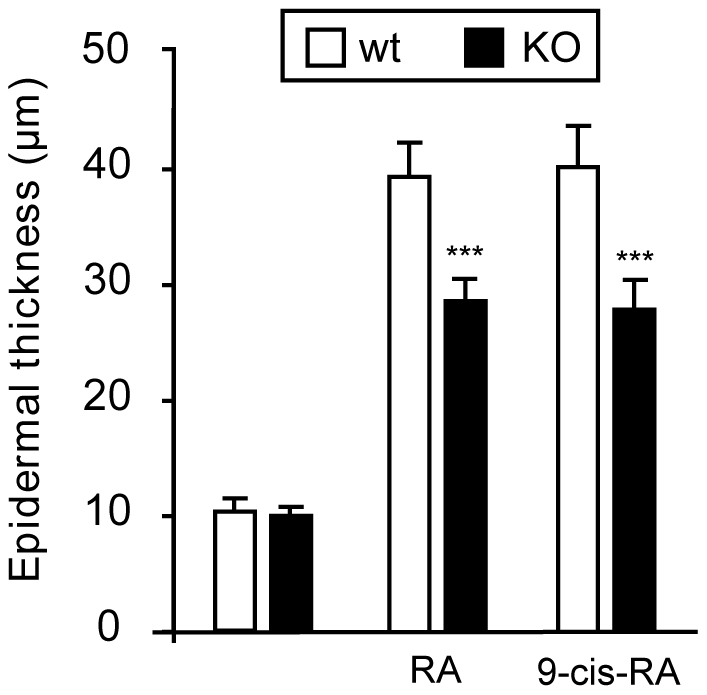
Reduced hyperplasia in response to retinoids in mice lacking TRs. Epidermal thickness was measured in wild-type and TR-deficient mice after topical treatment with all-trans-RA (ATRA) and 9-cis-RA. Data are shown as mean values ± SE, and ***(*P*<0.001) represents statistically significant differences relative to Wt mice.

We have previously observed that the skin of knock-out mice lacking TRα1 and TRβ, the major TR isoforms that bind thyroid hormones, shows reduced hyperplasia in response to topical treatment with the tumor promoter TPA. To extend our understanding on the role of TRs in skin proliferation, we investigated the response of these mice to 9-cis-RA that also causes epidermal proliferation [10]. As shown in Fig. 1A, the retinoid caused a significant increase in skin thickness in wild-type and KO mice, but KO animals displayed a reduced hyperplasia. As expected, a reduced response to TPA stimulation was also found in mice lacking TRs. To analyze if the reduced hyperplasia in these animals is a consequence of reduced keratinocyte proliferation, BrdU incorporation was examined in paraffin sections of 9-cis-RA and TPA-treated skin incubated with an anti-BrdU antibody. As shown in Fig. 2B, the BrdU labeling index increased significantly after treatment with both compounds and this response was also strongly reduced in KO mice. Fig. 1C shows representative images of the histology and BrdU incorporation found in skins treated with 9-cis-RA and TPA. It can be observed that although in wild-type mice both agents caused hyperplasia and increased BrdU incorporation, the retinoid (but not TPA) also produced parakeratosis [31], characterized by the retention of nuclei in the stratum corneum. Remarkably, in TR KO mice parakeratosis was much less common and was only observed in isolated areas. In addition, topical 9-cis-RA application caused the appearance of skin wounds, which were more frequent in mice lacking the receptors (data not shown).

To further analyze the role of TRs on the response to retinoids, we compared the hyperplasia obtained after topical application of 9-cis-RA and all-trans-RA in wild-type and KO mice. As shown in Fig. 2, both retinoids induced a similar increase in epidermal thickness and the response to both compounds was reduced to a similar extent in the animals lacking TRs. Since all-trans-RA does not bind RXR, these results confirm the implication of RAR in the epidermal hyperproliferative response.

**Figure 3:**
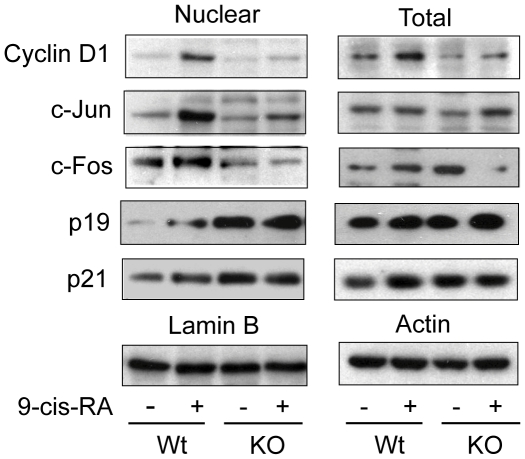
Altered expression of proliferation-related proteins in the skin of TR-deficient mice after 9-cis-RA treatment. Total and nuclear skin cell lysates from Wt and KO mice treated topically with 9-cis-RA were used for Western blotting with the indicated antibodies. Actin and lamin B were used as a loading control for total and nuclear extracts, respectively.

To analyze the mechanisms by which a reduced proliferative response to 9-cis-RA was found in TR KO mice, we measured by western blot the levels of proteins important for skin proliferation (Fig. 3). We have previously reported that induction of Cyclin D1 in response to TPA was strongly reduced in these animals [31], and as illustrated in Fig. 3, 9-cis-RA caused a strong induction of total and nuclear Cyclin D1 expression in wild-type but not in TR KO mice. Moreover, the nuclear levels of the AP-1 components c-Jun and c-Fos were significantly induced by the retinoid in normal mice but not in TR deficient mice. In addition, we analyzed expression of the cyclin kinase inhibitor (CKI) p21 and p19^arf^. As shown in Fig. 3, the nuclear levels of p19^arf^ were significantly higher in TR KO mice, and a less marked increase of p21 was also observed. In contrast with the changes found in the nuclear levels of these proteins, the changes observed in their total levels were modest, indicating that TRs can participate in regulating their cellular localization. The pattern of nuclear expression of the analyzed proteins could account for the skin hypoplasia observed in TR-deficient animals, since they play an important role in keratinocyte proliferation [32], [33].

### Hypothyroidism reduces 9-cis-RA-induced skin proliferation

**Figure 4:**
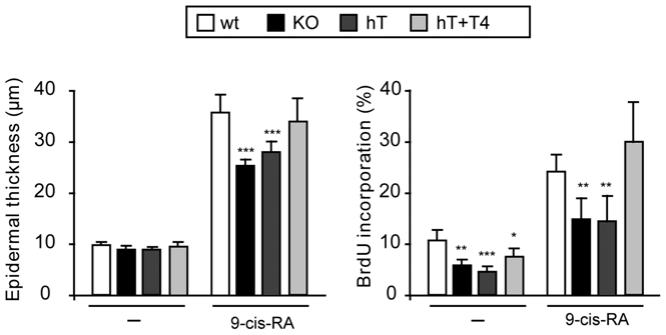
Hypothyroid mice and TR-deficient mice present similarly reduced proliferation in response to 9-cis-RA. Epidermal thickness (left panel) and BrdU incorporation (right panel) were determined in TR KO mice, mice treated with MMI and perchlorate for 4 months (hT) and in a group of animals receiving the anti-thyroidal drugs plus thyroxine (hT+T4).

We have previously shown that the response to TPA was similarly reduced in TR KO mice and in mice made hypothyroid by treatment with anti-thyroidal drugs. We then compared the response to 9-cis-RA in these animals. As shown in Fig. 4, hypothyroidism reduced epidermal hyperplasia and BrdU incorporation in response to the retinoid to an extent similar to that obtained in the mice lacking the receptors. Furthermore, the effect of hypothyroidism on epidermal thickness and proliferation was reversed when mice were treated with a physiological substitution dose of the thyroid hormone thyroxine, demonstrating that the observed effects are directly attributed to the thyroidal status and not to unspecific actions of the anti-thyroidal drugs used.

### Role of TRs in expression of epidermal markers

**Figure 5:**
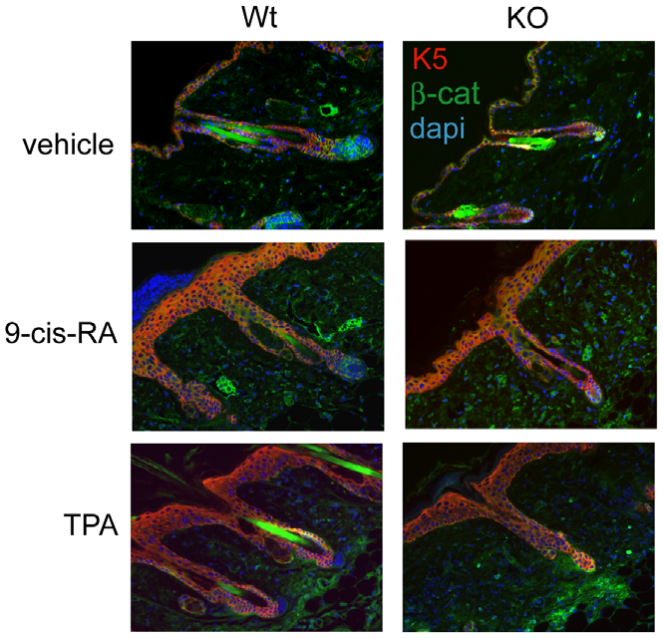
Expression of the proliferation marker keratin 5 is reduced in TR KO mice in response to proliferative stimuli. Examples of double immunofluorescence images of keratin 5 (K5, red) and β-catenin (green) expression in dorsal skin of wild-type (Wt) and TR-deficient mice (KO) after topical treatment with vehicle, 9-cis-RA or TPA. The slides were counterstained with dapi (blue) and the merged images are shown.

Keratins are used as epidermal markers due to their specific localization in the diverse skin layers. In normal skin K5 is expressed in the basal layer and in the hair follicle keratinocytes [34]. In agreement with our previous observations [31], K5 detected by immunofluorescence showed a normal expression and localization in vehicle-treated wild-type and KO mice. After 9-cis-RA or TPA treatment K5 expression is expanded in both groups, although the number of labeled keratinocyte layers was reduced in the KO animals, corresponding to the reduced hyperplasia (Fig. 5). Since the reduced proliferation in the intestinal epithelial cells of TRα-deficient mice has been related to changes in β-catenin [35], we also analyzed the expression and distribution of this protein in the skin. However, we did not find any significant change in β-catenin expression and/or localization in the absence of TRs.

**Figure 6:**
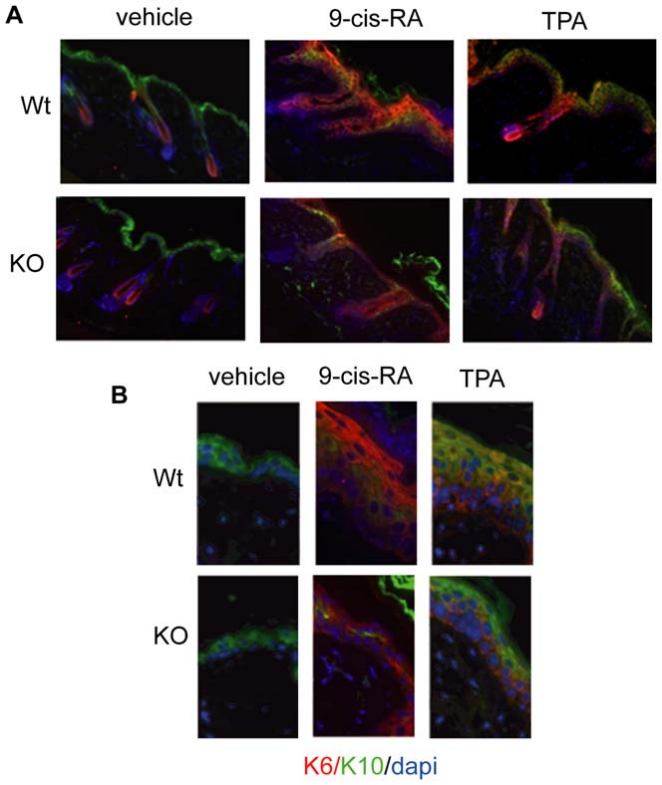
Keratin 6 and 10 expression in TR-deficient mice. A) The proliferation marker keratin 6 (K6, red) and the early differentiation marker keratin 10 (K10, green) were detected by immunofluorescence in wild-type and TR KO mice before and after application of 9-cis-RA or TPA. Nuclei were stained with dapi (blue). Panel B) represents a larger magnification of the merged images shown in A) for a better observation of K6 and K10 colocalization and the decrease in K10 expression observed in TR-deficient mice after 9-cis-RA treatment.

Under normal conditions K6 is only expressed in the keratinocytes of the hair follicle both in wild-type and KO animals. Topical application of 9-cis-RA extended K6 expression to upper keratinocyte layers of interfollicular epidermis in control mice and this increase was reduced in KO animals. TPA treatment also expanded K6 expression and there was a slight reduction of K6 in the more external keratinocyte layers (Fig. 6A). On the other hand, K10 is present in the stratum spinosum and is considered as an early differentiation marker of keratinocytes [36]. This keratin had also a normal distribution in vehicle-treated KO mice. TPA application induced the expansion of cell layers expressing K10 in the skins of both experimental groups in agreement with the induced hyperplasia, although less markedly in KOs. On the contrary, treatment with the retinoid reduced expression of K10 in wild-type mice, showing a discontinuous pattern with absence of labeling in some areas. This reduction was stronger in the skin of TR KO mice, suggesting that TRs deficiency accentuates the decrease in early differentiation of keratinocytes caused by the retinoid (Fig. 6A). As it can be observed in the magnifications shown in Fig. 6B, colocalization of K6 and K10 revealed marked differences between both groups and treatments. After TPA treatment, co-localization was found in the more external layers of the interfollicular epidermis in the wild-type mice, whereas in the KO mice co-localization was not observed due to the low expression of K10 in those layers. After 9-cis-RA treatment, and due to the reduction of K10 expression, colocalization of K6 and K10 was rarely observed in both experimental groups, being limited to small cellular groups in wild-type animals or even to isolated cells in KOs.

**Figure 7:**
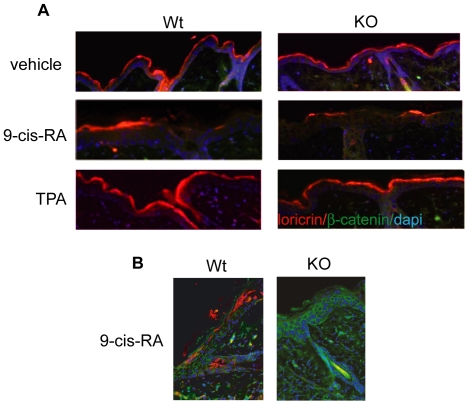
TR deletion alters expression of loricrin. A) Localization by immunofluorescence of the terminal differentiation marker loricrin in the most external epidermal layer of skins from wild-type and TR KO mice treated with 9-cis-RA and TPA. Loricrin expression (red) demonstrates that 9-cis-RA (but not TPA) promotes altered differentiation and that this is more marked in mice lacking TRs. Panel B) shows that loricrin is totally absent in large areas of the skin of some TR KO animals after 9-cis-RA treatment.

Loricrin is used as a marker for terminal differentiation, since it is expressed in the more external epidermal layer. This protein is synthesized in the stratum granulosum and is included in the cellular envelope of the keratinocytes in the stratum corneum [8], [37]. Loricrin expression and localization was normal in wild-type and KO animals under normal conditions and after TPA treatment (Fig. 7A). However, 9-cis-RA treatment reduced loricrin expression [8], which as in the case of K10 presented a patched discontinuous pattern. This reduction was again more prominent in the animals lacking TR (Fig. 7A) and in some animals loricrin expression disappeared in large areas (Fig. 7B). These results suggest that in the absence of TRs both early and late keratinocyte differentiation are further repressed upon retinoid treatment.

### Retinoid-induced inflammation is enhanced in the absence of TRs

**Figure 8:**
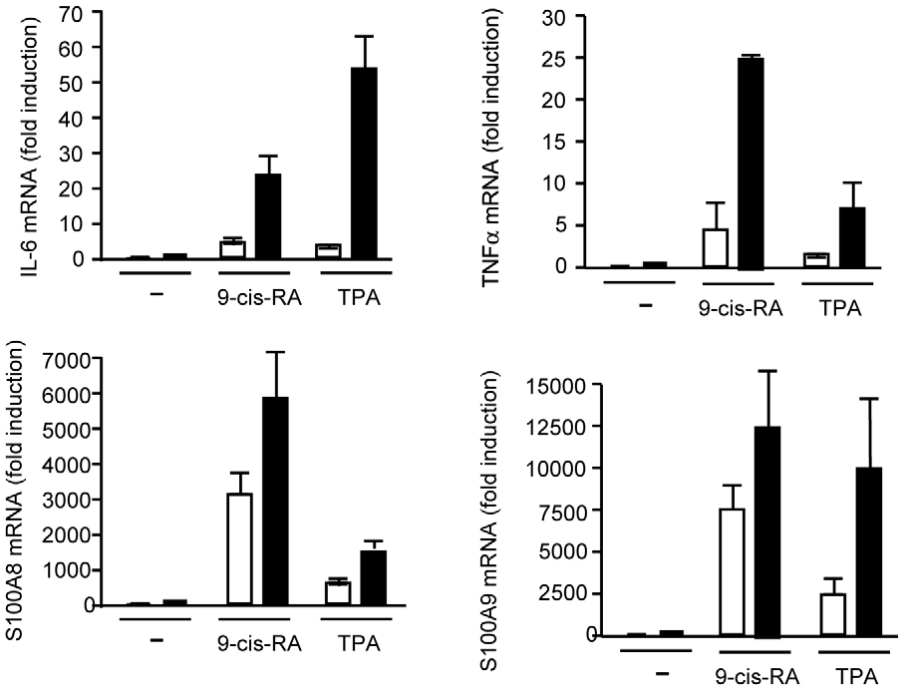
Increased expression of proinflammatory and chemotactic molecules in skins of TR KO mice. Relative mRNA levels of TNFα, IL-6, S100A8 and S100A9 in wild-type and TR KO mice treated with vehicle, 9-cis-RA or TPA. Values representing expression levels of vehicle-treated wil-type mice were set to 1, and fold induction is represented relative to this basal level.

We have observed that the skin inflammatory response to TPA is enhanced in TR KO mice, with an increase in the content of chemical mediators and augmented infiltration of immune cells. Since hyperproliferation in response to 9-cis-RA is also accompanied by inflammatory cells infiltration [10], [11], we measured mRNA levels of the proinflammatory cytokines IL-6 and TNFα and the chemotactic factors S100A8 and S100A9, in response to 9-cis-RA and TPA in the skin of wild-type and TR KO mice. As shown in Fig. 8, treatment with both compounds induced substantial increases in the transcripts coding for these proteins, which play pivotal roles in the recruitment of inflammatory cells, and this induction was stronger in the animals lacking the receptors.

**Figure 9:**
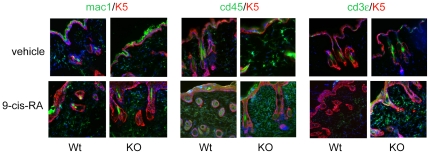
Immune cell infiltration is enhanced in skins of TR deficient mice treated with 9-cis-RA. Double immunofluorescence images of keratin 5 (K5) with mac-1 (left panel), cd45 (middle panel) and cd3ε (right panel) in skins from wild-type and TR KO mice treated with 9-cis-RA or vehicle alone. Nuclei were stained with dapi.

Immune cell infiltration in response to 9-cis-RA was analyzed by immunofluorescence with specific cell markers (mac1 for macrophages, cd45 for B and T lymphocytes and cd3ε for T lymphocytes). As shown in Fig. 9, immune cell infiltration was low in vehicle-treated mice, but increased significantly after the topical treatment with the retinoid, being more noticeable in the case of T lymphocytes, in the TR KO animals. These results reinforce the idea that TRs act as endogenous inhibitors of skin inflammation.

## Discussion

We have previously shown that TRs are required to attain a normal proliferative skin response to TPA, and our present results indicate that the response to retinoids is also regulated by these receptors. Retinoids are known to induce epidermal hyperplasia and our results show that this response is blunted in genetically modified mice lacking TRs. RA-induced skin hyperplasia most probably involves RARγ/RXRα heterodimers, in which RXR transcriptional activity is subordinated to that of RAR, i.e. liganded RXR is inactive unless its RAR partner is itself liganded [38]. Our data are compatible with this hypothesis, since lack of TRs affected both the response to 9-cis-RA and to the RAR-specific ligand all-trans-RA. As cell proliferation only takes place in the basal layer, the finding that RARγ/RXRα heterodimers, which are present in suprabasal keratinocytes, are required for RA-induced epidermal hyperplasia demonstrates that retinoids induce, through these heterodimers, the synthesis of a paracrine signal in suprabasal keratinocytes, which in turn causes hyperproliferation of basal keratinocytes [39], [40]. Therefore, TRs could modulate either the RA-induced signal or the response of the basal layer keratinocytes to that signal. Our results showing that the response to other hyperproliferative stimulus such as TPA, which act through different signalling pathways, is also affected in TR KO mice suggests that the second possibility is more likely. On the other hand, deletion of TRs, which share the heterodimeric partner with RARs, could favor formation of RXR homodimers, RAR/RXR heterodimers, or even heterodimerization of RXR with other nuclear receptors that could affect epidermal proliferation [41], [42]. However, the altered skin response to TPA is most likely independent of these mechanisms. In addition, we cannot dismiss the possibility that in the absence of TRs increased availability of coactivators or corepressors could affect the response to other nuclear receptors [43], or even other transcription factors that associate with those coregulators affecting skin physiology. Moreover, TR deficiency could also have indirect effects on the regulation of the skin response to retinoids or TPA. It is well known that thyroid hormones in mammalian cells affect basal metabolic rate, metabolism of nutrients, oxygen consumption and many other physiological processes that could regulate skin homeostasis in an indirect manner. For example, hypoxia, mitochondrial damage etc., could have similar effects as TR deficiency, blunting the proliferative responses to retinoids or TPA, while enhancing the proinflammatory responses evoked by the damaged cells. However, at least part of the effects of TRs appear to be exerted directly in the keratinocytes, since we have found decreased proliferation and increased inflammatory mediators in primary cultures of keratinocytes obtained from newborn TR KO mice [30].

It has been previously observed that the phenotype of hypothyroid mice is not identical to that of mice lacking TRs, and that some alterations can be even less severe in the latter [44], [45], [46], a difference that could be attributed to the effects of the unliganded receptors that can act as constitutive repressors [15], [47]. This does not appear to be the case with skin proliferation, since epidermal proliferation was similarly reduced in hypothyroid and TR KO animals not only after retinoid treatment, but also after application of TPA. Therefore, thyroid hormone binding to the receptor appears to be required for a normal proliferative response of epidermal keratinocytes. An additional factor to consider is the possible participation of thyrotropin (TSH) in the skin phenotype of hypothyroid and TR KO mice. Both groups of animals present high circulating levels of TSH and this hormone has been recently shown to influence skin physiology [48], [49]. However, the observation that topical application of the thyroid hormones stimulates epidermal proliferation in mice and rats [28], [50], together with our results obtained in primary keratinocytes favors the hypothesis of a direct role of TRs on keratinocyte proliferation.

We have previously shown that the decreased proliferative response of TR-deficient mice to TPA was accompanied by a strong reduction in the induction of Cyclin D1 [30], and we now also observe a markedly blunted Cyclin D1 response to topical retinoid application in these animals. Since Cyclin D1 is a key protein for keratinocyte proliferation [32], this reduction could play an important role in the observed phenotype. Furthermore, we confirmed a strong increase in the levels of the p19^arf^ in the skin nuclei of the TR KO mice, and we found lower levels of the AP-1 components c-Fos and c-Jun after retinoid treatment in these animals. These changes are also compatible with the reduced hyperplasia observed in TR-deficient animals. Interestingly, whereas the nuclear levels of c-Fos and c-Jun are reduced after retinoids administration in mice lacking TRs, these proteins were higher in these animals than in wild-type animals after TPA treatment [30], indicating again that different pathways underlie the proliferative defect found after TPA and 9-cis-RA treatments.

It has been reported that TRs can regulate either positively or negatively the expression of selected keratins in cultured cells. Whereas the thyroid hormone stimulates K15 promoter activity in transient transfection assays [51], K5 and K6 promoter activity is enhanced by unliganded TR and the hormone reverses this activation. This regulation appears to be mediated by a negative TRE located relatively close to the TATA box that can also mediate regulation by RA [52], [53], [54]. Our in vivo results indicate that the reduced epidermal proliferation after retinoid or TPA application was also evident by the changes in the expression of K5 and K6 in TR deficient mice. Although distribution of K5 was normal, a clear reduction in the number of cell layers expressing this marker was observed after treatment in these animals. With respect to K6, TPA treatment expanded K6 expression to suprabasal layers of the interfollicular epidermis, both in the presence and absence of TRs, as corresponding to a hyperproliferative state [55], [56]. However, reduced expression of K6 in the most external layers was observed after TPA treatment in the animals lacking TRs, a finding even more patent after treatment with 9-cis-RA. Although this reduction can be linked to the decreased epidermal proliferation in these animals, it has been described that K6 expression is regulated by thyroid hormones in cultured cells [41], and therefore a direct effect of TRs on this reduction cannot be discarded. Furthermore, the decreased K6 expression could be related to the reduced epidermal proliferation in TR KO mice since this keratin is known to increase epidermal growth factor receptors signaling pathways [57].

Expression of the early (K10) and late (loricrin) differentiation markers was not altered under basal conditions in the absence of TRs and TPA treatment did not interfere with epidermal differentiation. In normal animals 9-cis-RA treatment caused parakeratosis, an abnormal keratinization of cells associated with the thinning or loss of the granular layer, whereas this condition was rarely observed in the KO mice suggesting that differentiation could be altered in the absence of TRs. We observed that 9-cis-RA treatment reduced expression of K10 and loricrin in normal and TR deficient mice, in agreement with previous results demonstrating that retinoids impair induction of differentiation markers in cultured keratinocytes [6], [56] and after topical treatment in vivo [9]. However, this decrease was notably accentuated in the animals lacking TRs. These results demonstrate that TRs act as endogenous inhibitors of the actions of retinoid receptors in epidermal differentiation and indicate that although in the absence of TRs skin differentiation can occur normally under ordinary conditions, these receptors are required to limit the deleterious effects of retinoids on inhibiting differentiation of the epidermis.

In parallel with the hyperproliferative response, both TPA and 9-cis-RA provoke a strong inflammatory reaction [10], [11], [58], [59], [60]. We have previously observed that in the skin of TR KO mice this reaction is exacerbated after TPA treatment, with activation of p65/NF-κB and Stat3 phosphorylation, augmented expression of chemokines and pro-inflammatory cytokines and increased dermal infiltration of immune cells [30]. An excessive inflammatory response was also observed in TRs deficient mice after 9-cis-RA treatment. mRNAs for the pro-inflammatory cytokines Il-6 and TNFα were more strongly induced in the TR KO mice, and the same occurred with the transcripts for the neutrophil recruiting proteins S100A8 and S100A9, which are also believed to play an important role in the pathogenesis of epidermal disease and inflammation [61]. The ability of several members of the nuclear receptor superfamily to attenuate inflammatory responses by transrepressing activation of inflammatory response genes is well known. This transrepression occurs by protein-protein interactions of the receptors with coregulatory proteins and promoter-bound transcription factors, rather than by direct, sequence-specific interactions with DNA [62]. Our results reinforce the idea that thyroid receptors can also act as endogenous controllers of inflammation, at least in the skin, limiting immune responses to various pro-inflammatory stimuli through repression of cytokines and chemotactic proteins.

In summary, our present results increase the knowledge of the role of TRs and thyroid hormones in the skin. The liganded TRs are required for a normal response of keratinocytes to retinoid-induced proliferation and can also modulate keratinocytes differentiation increasing the ability of these compounds to block both early and terminal differentiation. Due to the relevant role of retinoids in skin physiological and their wide therapeutic effects, modulation of the responses to these agents by the thyroid hormone receptors could have important functional implications.

## Materials and Methods

### Animals and Treatments

All animal work was done in compliance with the European Community Law (86/609/EEC) and the Spanish law (R.D. 1201/2005), with approval of the Ethics Committee of the Consejo Superior de Investigaciones Científicas. Experiments were performed in adult female mice. TRα1^−/−^/TRβ^−/−^ double knockout (KO) mice and wild-type (TRα1^+/+^/TRβ^+/+^) animals with the same background [63] were genotyped and used for the studies. Double KO males were crossed with heterocygote females, and back-crosses were performed to avoid genetic drift. Wild-type mice were made hypothyroid (hT) by treatment with 0.02% methymazole and 0.1% sodium perchlorate in the drinking water [20], [30]. Treatment started when animals were 1 month old and was continued for 4 months. This treatment reduced serum levels of T4 measured with an ECL-based kit (Diagnostic Products Corp. Los Angeles, CA) by approximately 80%. Starting one week after treatment with the anti-thyroidal drugs, a group of mice received daily a physiological dose of thyroxine (T4, 20 ng/g body weight) (hT+T4). Dorsal skins were shaved and treated with depilatory cream 24 h before the treatments with retinoids or TPA. The retinoids 9-cis-RA and all-trans-RA (15 µg/mouse) were applied topically once a day for 4 days and mice were killed 24 h after the last application. TPA was applied twice (at days 2 and 4) and animals were sacrificed at day 5. In the control group, animals were treated with vehicle (acetone) only. At the end of the experiments, skin was excised and either frozen to obtain RNA or proteins, or fixed with 4% paraformaldehyde or 70% ethanol and embedded in paraffin. Between 4 and 6 animals/experimental group were used in each experiment. Skin sections were stained with hematoxilin and eosin (H&E) or processed for immunohistochemistry or immunofluorescence. For frozen sections (5 µm), fresh skin samples were embedded in OCT (TissueTech) and kept frozen (−80°C) until use.

### Morphometric Analysis

Quantitation of the epidermal thickness was performed in dorsal skin sections stained with H&E. At least 10 individual fields of 1 mm per slide from five mice of each experimental group were counted using the software MetaMorph (Premier Offline 7.0; Molecular Devices, Downingtown, PA).

### BrdU Labeling

Epidermal proliferation was determined after ip injection of bromodeoxyuridine (BrdU; 0.1 mg/g weight), 1 h prior sacrifice. BrdU incorporation was detected by immunohistochemistry using standard protocols on deparaffinized sections. Immunohistochemistry was performed with mouse anti-BrdU monoclonal antibody (Roche, no. 1170376) as previously described, and the percentage of BrdU-positive per total basal keratinocytes was calculated [30], [64].

### Immunofluorescence

Epidermal differentiation was analyzed by indirect immunofluorescence in frozen sections of skin as previously described [65], [66]. Rabbit polyclonal antibodies antibodies against K5, K6, and loricrin were obtained from Covance (Princeton, New Jersey 08540, USA), and used at 1∶500 or 1∶1000 dilutions. Keratin K10 was detected using a monoclonal antibody (Sta Cruz Biotechnology, Santa Cruz, CA) at a 1∶100 dilution. Immunodetection of dermal inflammatory cells including T lymphocytes (anti-CD3ε), T and B lymphocytes (anti-CD45), and macrophages (anti-CD11b/Mac1) was performed using fluorescein isothiocyanate-labeled specific rat anti-mouse monoclonal antibodies (BD Pharmigen, 10975 Torreyana Road, San Diego, CA 92121) at 1∶50 dilution. Secondary antibodies were obtained from Jackson ImmunoResearch Laboratories, Inc. (Suffolk, England) and were used as previously described [66].

### Western blot

Whole-cell and nuclear extracts [30] were isolated from the skin of wild-type and TR KO mice treated with acetone or 9-cis-RA. Samples from 4 individual animals in each group were pooled, and 50 µg of proteins were subjected to polyacrylamide gel electrophoresis, transferred to nitrocellulose membranes and used for western blot as previously described [30]. Antibodies to Cyclin D1 (sc- 718), c-Jun (sc-1694), c-Fos (sc-52), lamin B (sc-6217), and actin (sc-1616) were obtained from Santa Cruz Biotechnology, Inc. (Santa Cruz, CA), and antibodies against p19^ arf^ (Ab-7960) and p21 (Ab-80) were purchased from Abcam Inc. (Cambridge, UK).

### Quantitative real-time PCR assays

Total RNA was extracted from skin using RNeasy Fibrous Tissues Mini Kit (Qiagen Iberia S.L, Las Matas, Spain) and mRNA levels were analyzed in triplicate samples by quantitative RT-Q-PCR as previously described [30], following specifications of SuperScript™ First-Strand Synthesis System (Invitrogen Life Technologies Carlsbad, California 92008, USA). The primers for used were: 5′-GAACTGGCAGAAGAGGCACT -3′ (forward) and 5′-AGGGTCTGGGCCATAGAACT-3′ (reverse) for TNFα; 5′-AGTTGCCTTCTTGGGACTG-3′ (forward) and 5′-CAGAATTGCCATTGCACAA-3′ (reverse) for IL-6; 5′-GGAATCACCATGCCCTCTA-3′ (forward) and 5′-TGGCTGTCTTTGTGAG ATGC-3′ for S100A8 and 5′-TCATCGACACCTTCCATCAA-3′ (forward) and 5′-GTCCTGGTTTGTGTCCAGGT-3′ (reverse) for S100A9. Data analysis was done using the comparative CT method and data were corrected with the GPDH mRNA levels estimated with primers 5′-ACACTGCATGCCATCACTGCC-3′ (forward) and 5′-GCCTGCTTCACCACCTTCTTG-3′ (reverse).
